# Aging Increases Compensatory Saccade Amplitude in the Video Head Impulse Test

**DOI:** 10.3389/fneur.2016.00113

**Published:** 2016-07-18

**Authors:** Eric R. Anson, Robin T. Bigelow, John P. Carey, Quan-Li Xue, Stephanie Studenski, Michael C. Schubert, Konrad P. Weber, Yuri Agrawal

**Affiliations:** ^1^Department of Otolaryngology-Head and Neck Surgery, Johns Hopkins University School of Medicine, Baltimore, MD, USA; ^2^Department of Medicine, Johns Hopkins University School of Medicine, Baltimore, MD, USA; ^3^Center on Aging and Health, Johns Hopkins Medical Institutions, Baltimore, MD, USA; ^4^Longitudinal Studies Section, National Institute on Aging, Baltimore, MD, USA; ^5^Department of Neurology, University Hospital Zurich, University of Zurich, Zurich, Switzerland; ^6^Department of Ophthalmology, University Hospital Zurich, University of Zurich, Zurich, Switzerland

**Keywords:** VOR, compensatory saccades, healthy aging, head impulse test, vestibular

## Abstract

**Objective:**

Rotational vestibular function declines with age resulting in saccades as a compensatory mechanism to improve impaired gaze stability. Small reductions in rotational vestibulo-ocular reflex (VOR) gain that would be considered clinically normal have been associated with compensatory saccades. We evaluated whether compensatory saccade characteristics varied as a function of age, independent of semicircular canal function as quantified by VOR gain.

**Methods:**

Horizontal VOR gain was measured in 243 participants age 27–93 from the Baltimore Longitudinal Study of Aging using video head impulse testing. Latency and amplitude of the first saccade (either *covert* – occurring during head impulse, or *overt* – occurring following head impulse) were measured for head impulses with compensatory saccades (*n* = 2230 head impulses). The relationship between age and saccade latency, as well as the relationship between age and saccade amplitude, were evaluated using regression analyses adjusting for VOR gain, gender, and race.

**Results:**

Older adults (mean age 75.9) made significantly larger compensatory saccades relative to younger adults (mean age 45.0). In analyses adjusted for VOR gain, there was a significant association between age and amplitude of the first compensatory covert saccade (β = 0.015, *p* = 0.008). In analyses adjusted for VOR gain, there was a significant association between age and amplitude of the first compensatory overt saccade (β = 0.02, *p* < 0.001). Compensatory saccade latencies did not vary significantly by age.

**Conclusion:**

We observed that aging *increases* the compensatory catch-up saccade amplitude in healthy adults after controlling for VOR gain. Size of compensatory saccades may be useful in addition to VOR gain for characterizing vestibular function in aging adults.

## Introduction

Several studies have shown that rotational vestibular function declines with age (1–3). There is a reduction in the quantity of vestibular hair cells that occurs as a consequence of aging (4–7). This may result in alterations in vestibular function, characterized by reduced vestibulo-ocular reflex (VOR) gain and increased VOR gain variability with advancing age (1, 2, 8). Reduced function of the angular VOR results in generation of compensatory saccades (3, 9, 10). Studies have shown that compensatory saccades occur more frequently with increasing age (3, 11). Interestingly, compensatory saccades have been observed in older individuals even when VOR gain was clinically in the normal range (10, 11).

It is unclear whether the increased frequency and amplitude of compensatory saccades with age reflect greater VOR gain deficits in older individuals, which may be either clinical or subclinical (12, 13). Moreover, it is unclear whether saccade characteristics in older adults, such as saccade timing and amplitude, differ from saccades observed in younger individuals.

In this study, we investigate the relationship between age and compensatory saccade amplitude and latency in a cohort of healthy older adults within the Baltimore Longitudinal Study of Aging (BLSA). We used video head impulse testing (vHIT) to measure horizontal semicircular canal function (quantified as VOR gain) and to detect compensatory saccades. We considered both covert saccades (occurring during the head impulse) and overt saccades (occurring after the head impulse), separately (14–16). The goal of the study is to evaluate whether aging influences compensatory saccade latency and amplitude or whether the compensatory saccades observed in older individuals correlate only with reduced VOR function.

## Materials and Methods

### Participants

The BLSA is an ongoing prospective cohort study initiated by the National Institute on Aging (NIA), in 1958. Participants are community-dwelling participants aged 20–103 who undergo a standardized array of tests over 3 days, every 1–4 years at the NIA. This study evaluated a cross-sectional sample of all BLSA participants between June 2014 and April 2015. During this time period, 243 participants completed vHIT testing. All participants provided written-informed consent, and the BLSA study protocol was approved by the Institutional Review Board at Harbor Hospital. Participants were asked to identify their race from the following options: White, Black or African-Americans, Asian, American Indian or Alaska Native, Native Hawaiian or Other Pacific Islander, “Two or More Races,” “Don’t Know,” or “Refused.” Race-ethnicity was grouped as “white,” “black,” and “other,” as the majority of participants were either white or black.

### Video Head Impulse Testing

Vestibular function was measured for the horizontal VOR using vHIT. Methods to measure horizontal semicircular canal function have been published previously and validated in older adults (8, 17–19). In brief, participants wore the EyeSeeCam video-oculography system, a lightweight goggle frame with a built in camera to record right eye movements and an accelerometer to record head movement at a sampling frequency of 220 Hz (Interacoustics, Eden Prairie, MN, USA). Participants sat approximately 1.25 m from a visual fixation target on the wall. Trained examiners tilted the participant’s head 30° below horizontal to bring the horizontal semicircular canal into the plane of head rotation and then performed 10–15 small amplitude (15–20°) “center-out” head impulses to the right and left, with peak velocity typically from 150 to 250°/s.

**Figure 1:**
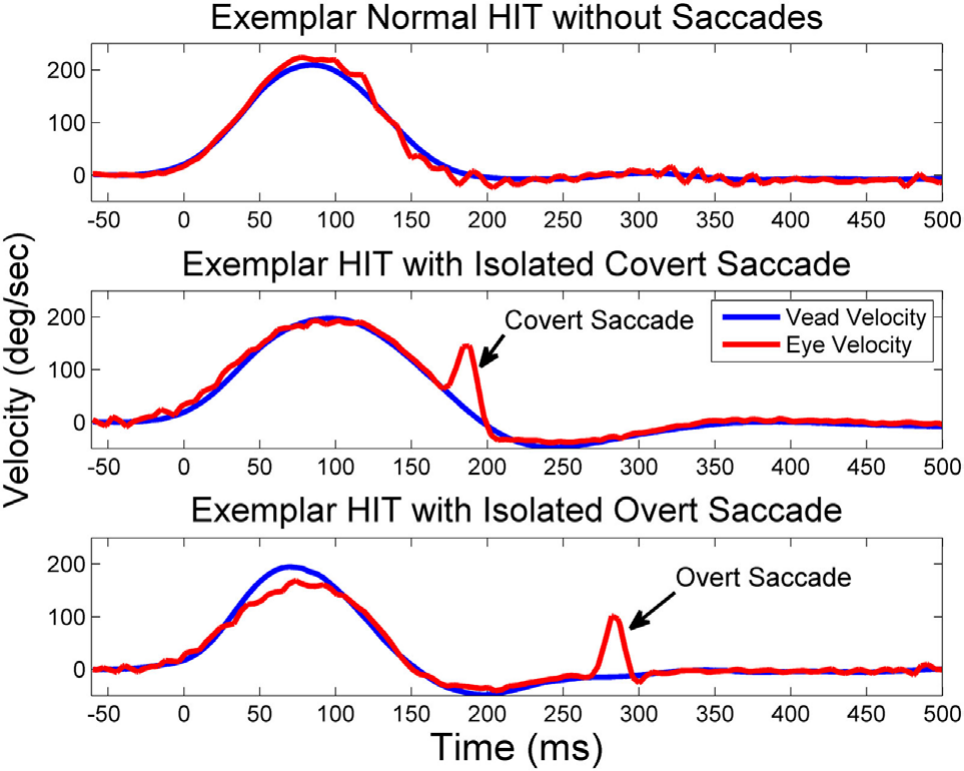
**Exemplar HITs with and without saccades**. Exemplar vHITs showing the presence of a normal response without saccades (upper pane), an isolated covert compensatory saccade (middle pane), and an isolated overt saccade (lower pane). Saccades are indicated by arrows. Eye velocity (red) has been inverted and shown superimposed on head velocity (blue).

The EyeSeeCam software provides interpolated head impulse data at 1000 Hz in the exported MATLAB data file, which we used for subsequent *post hoc* analyses. During *post hoc* analysis, experienced evaluators examined individual head impulse traces using custom software (MATLAB, MathWorks) and rejected head impulses that had pupil tracking artifact during the head impulse or incorrectly performed head impulses (i.e., low peak head velocity, excessive head recoil, or overshoot) (20). Horizontal VOR gain was calculated as the ratio of the area under (AUC) the de-saccaded eye velocity curve over the area under the head velocity curve from the onset of the head impulse until head velocity returned to 0 (21). This method of gain calculation has been shown to be least susceptible to methodological errors (20). Saccades were initially identified by an automatic detection algorithm based on eye accelerations greater than 4000°/s^2^ and visually verified by experienced examiners. Covert saccades were defined as any saccade starting before head velocity returns to 0, and overt saccades were defined as any saccade starting after head velocity returns to 0, see Figure 1 (15). In order to simplify the regression analysis, head impulses without any saccades were excluded from analyses relating age to amplitude and latency of the first compensatory saccade and were not used to calculate VOR gain. Head impulses without saccades were included in the normalization of the cumulative saccade amplitude to reduce the potential for sample bias. Head impulses with bi-directional saccades were also excluded from these analyses.

**Figure 2:**
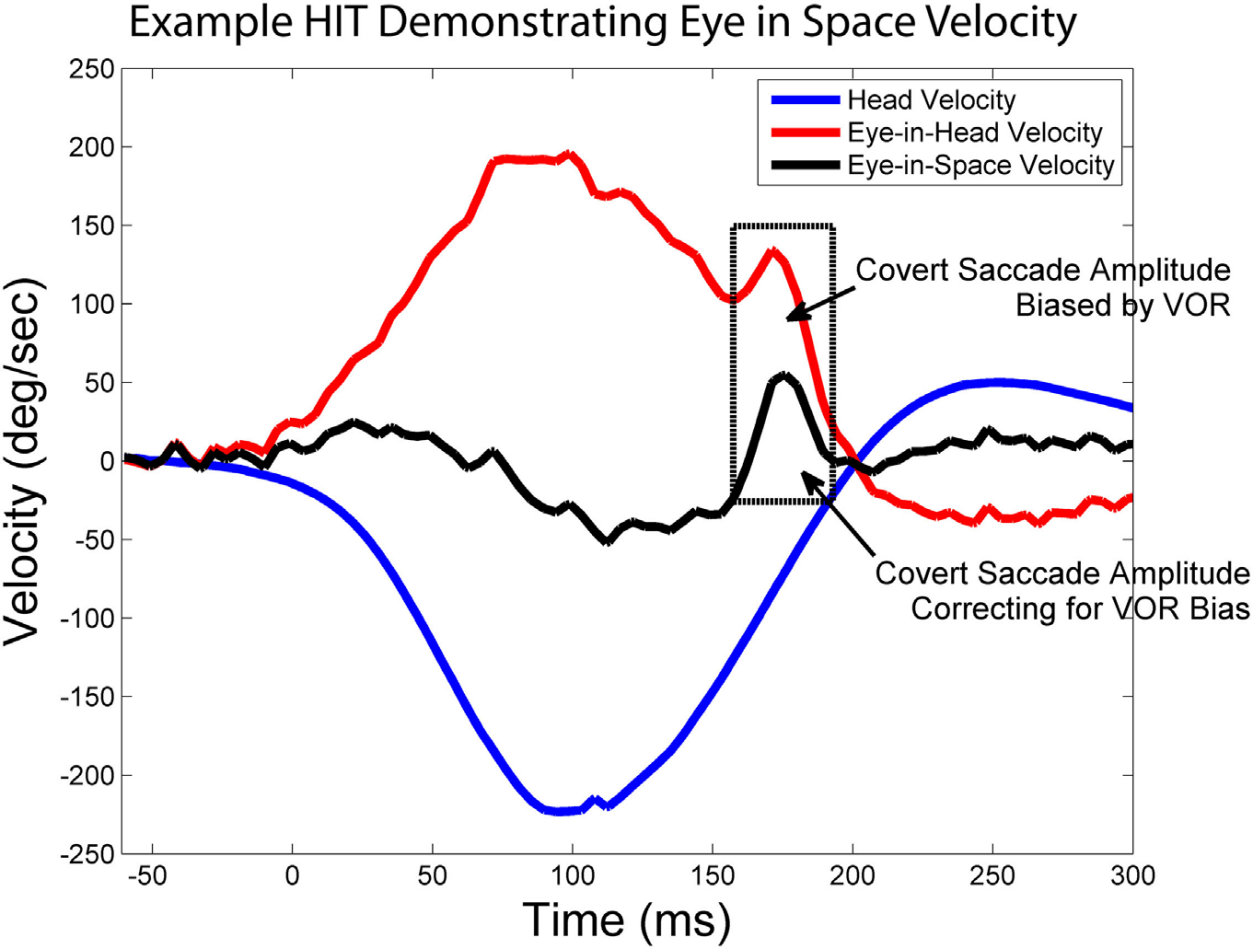
**Calculating eye-in-space velocity**. Exemplar vHIT showing how the sum of head velocity (blue) and eye-in-head velocity (red) can be used to calculate eye-in-space velocity (black). The boxed in area highlights a covert saccade and demonstrates how using eye-in-space rather than eye-in-head velocity to calculate saccade amplitude removes the bias that results from VOR-mediated eye movement.

To exclude volitional saccades (i.e., saccades not generated reflexively to compensate for a deficient VOR), we only analyzed saccades when the saccade latency was between 25 and 503 ms after head impulse testing (HIT) onset, defined as head velocity >20°/s (22). We chose 25 ms as the earliest that saccades may be present based on previously reported ranges for both active and passive HIT (23). To determine the latest time period for non-volitional compensatory saccades, we first estimated the population average HIT duration from our sample by bootstrapping 1000 new populations allowing for resampling, with 500 nested resamples for variance estimates (24). This resulted in a population average HIT duration of 253 ms. We identified 250 ms after the HIT ended (503 ms) as the criteria after which saccades could not be reliably attributed to the HIT stimulus based on reported values for volitional saccade latencies in older adults (25, 26). Saccade latency (time from the onset of the HIT until the onset of the saccade) and amplitude for the first compensatory saccade was determined for each HIT with a compensatory saccade. Covert saccade amplitude was adjusted such that the velocity of the VOR and the resultant position change of the eye due to the VOR were removed. Eye velocity was summed with head velocity to calculate the velocity of the eye in space (because both velocities followed the convention that movement to the right was positive, to the left was negative), see Figure 2. Amplitude for all saccades was calculated as the area under the curve for the saccade based on the eye-in-space velocity. This served to remove the VOR component from covert saccades, and the amplitudes were largely unchanged for overt saccades since the head was not moving during those saccades.

### Data Analysis

We combined rightward and leftward HITs for a total sample of 486 ears, of which 452 had at least one head impulse that resulted in a compensatory saccade. We plotted cumulative saccade amplitude as a function of saccade latency across the cohort, to graphically represent the population of saccades observed in the cohort and differences between younger (<60 years, mean age 45.0) and older (≥60 years, mean age 75.9) age groups. Cumulative saccade amplitudes in latency bins of 10 ms were normalized to account for the number of individuals and head impulses with and without compensatory saccades in each bin. *T*-tests were used to compare mean saccade amplitude and latency between young and older adults, separately for overt and covert saccades. Analyses of saccade latency and amplitude were based on individual head impulses with compensatory saccades [*n* = 2223 (353 head impulses with covert saccades, 1870 head impulses with overt saccades), representing 227 participants]. All analyses accounted for clustering by individual using mixed effects regression models. Covert vs. overt compensatory saccades were evaluated in separate regression models. We evaluated the association between latency of the first saccade and age and the association between amplitude of the first saccade and age in multivariate analyses adjusted for VOR gain, gender, and race. We included the individual-specific mean head impulse parameters (i.e., VOR gain, saccade latency, amplitude) as random effects to account for the within-individual correlation between the repeated head impulses. This results in valid statistical inference for the fixed effects (i.e., age, gender, and race). We estimated the model parameters *via* maximum likelihood using xtmixed in STATA. All mixed effects regression models were adjusted for age, gender, and race. We adjusted alpha levels within specific questions: for regression analyses α = 0. 0125 (applying a Bonferroni correction for four regressions), and an adjusted α = 0.0125 (applying a Bonferroni correction for four comparisons) was used for *t*-test comparisons.

## Results

The mean age of participants was 72.3 (SD 16.4, range 27–93) and 52% of the participants were female. Sixty-two percent of the participants were white, 21% of the participants were black, and the remaining 17% were classified as other. One hundred seventy-four of 486 ears (35.8%) had at least one head impulse with a covert saccade, and 417 of 486 ears (85.8%) had at least one head impulse with an overt saccade. The mean peak head velocity during head impulses with saccades was 212.9°/s (SD 31.4°/s).

**Figure 3:**
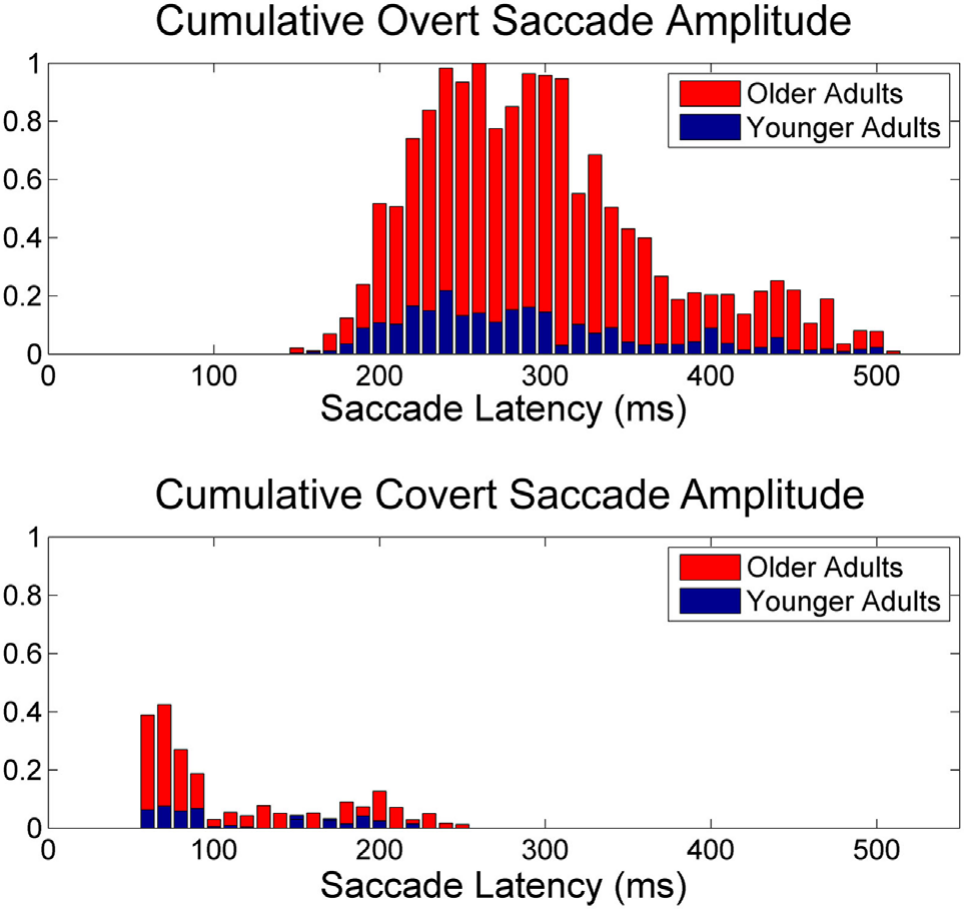
**Normalized saccade amplitude**. Normalized cumulative saccade amplitude for overt (upper panel) and VOR corrected covert (lower panel) saccades as a function of latency following onset of head impulse. Note the normalized cumulative amplitude is larger for older adults (≥60 years) relative to younger adults (<60 years) and that overt saccades have larger amplitude compared to covert saccades after correcting for VOR eye velocity and amplitude.

The normalized cumulative amplitude of overt and covert saccades as a function of saccade latency was plotted for younger (*n* = 28) adults (<60 years old) and older adults (*n* = 199; Figure 3). Mean amplitudes for overt saccades were significantly larger for older vs. younger adults (Table 1). Mean latencies were not significantly different for older vs. younger adults (Table 1).

**Table 1:** **Mean (SD) compensatory (overt, covert) saccade latency and amplitude for younger and older adults**.

	Younger adults (age 45.0, *N* = 28)	Older adults (age 75.9, *N* = 199)	*t*-test *p* value
**Overt**			
Mean latency (ms)	298.2 (86.1)	302.2 (74.3)	0.4834
Mean amplitude (deg)	1.2 (0.8)	1.7 (1.4)	0.0000*
**Covert**			
Mean latency (ms)	96.1 (52)	87.3 (49.5)	0.2949
Mean amplitude (deg)	1.1 (0.8)	1.4 (1.1)	0.1042

Significant results indicated by * for p < 0.0125.

### Relationship between Saccade Metrics and Age

#### Covert Saccades

We evaluated whether the covert saccade amplitudes were correlated with differences in VOR gain between older and younger adults or whether age may be an independent contributor (Table 2). In multivariate analyses adjusting for VOR gain, we observed a persistent significant association between age and amplitude of the first compensatory covert saccade (β = 0.015, *p* = 0.008). This association corresponds to an increase in covert saccade amplitude of approximately 0.15° for every decade of life, while controlling for VOR gain. There was no significant association between age or VOR gain and latency of the first compensatory covert saccade (Table 2).

**Table 2:** **Relationship between latency and amplitude of the first covert saccade and age controlling for VOR gain**.

	Covert saccade latency			Covert saccade amplitude		
Predictor variables	β	*p*	95% CI	β	p	95% CI
Age	−0.03	0.942	(−0.75, 0.70)	0.015	0.008*	(0.004, 0.026)
VOR gain	34.04	0.055	(−0.79, 68.9)	0.47	0.237	(−0.31, −1.25)
**Race**						
White	Ref	Ref	Ref	Ref	Ref	Ref
Black	−25.49	0.038	(−49.51, −1.47)	0.015	0.935	(−0.35, 0.38)
Other	−12.26	0.309	(−35.86, 11.34)	0.30	0.091	(−0.05, 0.65)
**Gender**						
Female	Ref	Ref	Ref	Ref	Ref	Ref
Male	−23.43	0.014	(−42.11, −4.76)	−0.30	0.038	(−0.59, −0.017)

Significant results indicated by * for p < 0.0125.

#### Overt Saccades

We next evaluated whether overt saccade amplitudes were correlated with differences in VOR gain between older and younger adults or whether age was an independent predictor (Table 3). In analyses adjusted for VOR gain, there was a significant association between age and amplitude of the first compensatory overt saccade (β = 0.02, *p* < 0.001). This association corresponds to an increase in overt saccade amplitude of approximately 0.2° for every decade of life, while controlling for VOR gain. There was no significant association between age and latency of the first compensatory overt saccade. Additionally, there was a significant association between VOR gain and amplitude of the first compensatory overt saccade (β = −4.03, *p* < 0.001). This association corresponds to a decrease in overt saccade amplitude of approximately 0.4° for every 0.1 increase in VOR gain. We also observed a significant association between VOR gain and overt saccade latency (β = 98.44, *p* < 0.001). This corresponds to overt saccades happening 10 ms later for every 0.1 increase in VOR gain.

**Table 3:** **Relationship between latency and amplitude of the first overt saccade and age controlling for VOR gain**.

	Overt saccade latency			Overt saccade amplitude		
Predictor variables	β	*p*	95% CI	β	p	95% CI
Age	0.189	0.512	(−0.37, 0.76)	0.020	0.000*	(0.011, 0.028)
VOR gain	98.44	0.000*	(66.0, 130.89)	−4.03	0.000*	(−4.47, −3.58)
**Race**						
White	Ref	Ref	Ref	Ref	Ref	Ref
Black	0.20	0.982	(−17.32, 17.72)	0.047	0.727	(−0.22, 0.31)
Other	−6.58	0.525	(−26.85, 13.69)	0.24	0.129	(−0.07, 0.55)
**Gender**						
Female	Ref	Ref	Ref	Ref	Ref	Ref
Male	1.71	0.818	(−12.84, 16.26)	−0.15	0.197	(−0.37, 0.076)

Significant results indicated by * for p < 0.0125.

## Discussion

The current results demonstrate that healthy older adults make significantly larger covert and overt compensatory saccades relative to younger adults. Additionally, we found that the higher amplitude compensatory saccades in older individuals were not fully explained by reduced VOR function in this group: even after accounting for lower VOR gain, older adults made significantly larger saccades. There are several potential explanations for these findings. First, the most straight forward explanation is that slight reductions in VOR gain with age are sufficient to trigger compensatory saccades. Second, it is possible that the saccade-generating mechanism in older adults may be impaired relative to younger adults. Increased levels of cerebellar disinhibition in older adults may contribute to reduced VOR calibration and generation of over-compensatory saccades (27). Maladaptive interaction between the paramedian pontine reticular formation and the vestibular nuclei may also contribute to the relationship between VOR gain and saccade amplitude reported here (28, 29). Although saccades triggered by target-driven head free gaze shifts were observed to be smaller and slower in older adults (30), compensatory saccades triggered by retinal slip or position error due to VOR insufficiency may result from a different neural mechanism or behave differently in the context of aging. Alternatively, declines in peripheral vestibular function in the context of aging may result not only in decreases in VOR gain but might also affect gaze stabilization by other mechanisms. Peng et al. showed that, during HIT, humans demonstrate a range of corrective eye movements superimposed on the VOR (31). Some of these eye movements correct primarily gaze velocity (i.e., retinal slip) errors and some correct primarily gaze position (i.e., foveal displacement) errors. The distribution of these corrective eye movements were found to depend on the status of vestibular function and the degree of challenge of the stimulus. In particular, normal humans exposed to high acceleration head impulses in their study employed more saccades that corrected gaze position errors late in the course of the head impulse after gaze velocity error was minimized. Thus, gaze stabilization during head impulses appears to rely not only on the VOR but on eye movements that correct for gaze position errors as well. Whether the distribution of these eye movements changes with age will require further research, but it is clear that VOR gain is only a partial measure of gaze stabilization ability.

In this sample, there were approximately 6× more overt saccades than covert saccades. Individuals recovering from acute surgical unilateral vestibular loss quickly begin using compensatory saccades that, over a period of days, shift earlier in time, eventually becoming covert in their timing (32). Some authors have hypothesized that covert saccades are generated in response to a combination of vestibular signals, gaze velocity error, and gaze position error, while overt saccades are thought to be generated in response to gaze position error (given that the head is still, so head and eye velocity are both equal to 0) (16, 33, 34). It is possible that gaze velocity error during the head impulses did not exceed 2–4°/s, typically tolerated by healthy adults (35, 36). The majority of individuals in this study had VOR gain close to unity, which suggests only minimal retinal slip throughout the head impulse, despite the presence of compensatory saccades. Alternatively, the disproportionate number of overt saccades suggests that the individuals in this study may have reduced ability to detect or quickly respond to gaze velocity errors that would produce covert saccades. The higher percentage of overt saccades may also have been an artifact of the less predictable “center-out” head impulses employed in this study (37). It is not clear whether saccadic adaptation to age-related decline in vestibular function – which is typically incomplete and bilateral – is different from that which occurs following complete vestibular loss, which is typically complete and unilateral. Determining whether compensatory saccades in older adults are preferentially generated in response to retinal position vs. retinal velocity errors will have implications for vestibular rehabilitation (38–40).

The current findings did not support any relationship between compensatory saccade latency and age, in contrast with previous work on discrete saccadic eye movements (25). The lack of age dependence for compensatory saccade latency is consistent with previous research, suggesting that compensatory saccade timing is a stereotypic, learned behavior (41). Latency of saccades during head impulses decreases and becomes less variable over time, during recovery from loss of vestibular function (32). Unlike individuals recovering from unilateral vestibular loss, the cross-sectional population of older adults in this study may be at different stages in compensation/adaptation for age-related vestibular loss. Prospective longitudinal studies would be necessary to demonstrate whether adults with age-related decline in vestibular function follow a similar learning profile with respect to latency of compensatory saccades.

We observed that the saccade amplitude is larger for older adults in a cohort of healthy adults and that saccade amplitude is in part related to VOR gain. It is interesting to note that, in this cohort, only 17 individuals had abnormally low VOR gain (VOR gain <0.68) as defined in previous studies (19). Future studies should determine whether saccade amplitude may provide a more complete picture of gaze stability in aging adults than VOR gain alone. Additionally, future studies should determine whether saccade amplitude is more strongly related to measures of functional mobility like balance and walking in older adults than VOR gain. Higher compensatory saccade amplitudes may contribute to gaze instability or oscillopsia with walking head motion, which may, in turn, result in slower walking speeds in older adults.

### Limitations

These data are cross-sectional and cannot be used to support causal inferences between changes in age and changes in compensatory saccades or VOR gain. Moreover, we cannot determine from these data whether the larger compensatory saccades in healthy older adults observed in this study represent adaptive or perhaps maladaptive compensation to change in rotational VOR function. We could not precisely measure eye position relative to a world-fixed target using the video head impulse system, which would be needed to determine whether the compensatory saccades brought the eye back to, or potentially overshot, the target. Measurement systems capable of quantifying the target location relative to the head are needed to address this limitation. We also only considered horizontal VOR gain in this study and future studies will need to establish whether these relationships hold for the vertical canals as well. Finally, to simplify our analysis and to ensure that we were capturing saccades of vestibular origin, we only considered the first compensatory saccade. A number of head impulses resulted in mixed types of saccades, and their significance will require future characterization and study.

## Conclusion

The size of compensatory saccades is larger for older adults compared to younger adults, even after accounting for level of similar rotational vestibular function as measured by VOR gain. The amplitude of compensatory saccades may be an additional indicator of decline in gaze stabilization ability in aging adults along with VOR gain.

## Author Contributions

EA and RB collected the data. EA, Q-LX, and YA drafted the manuscript. EA, Q-LX, and YA conducted the statistical analysis and interpreted the statistical analysis. EA, RB, JC, MS, SS, KW, and YA interpreted the data and critically edited the manuscript. YA designed the experiment. All authors approved the submitted version of the manuscript and are accountable for the accuracy and integrity of the work.

## Conflict of Interest Statement

The authors declare that the research was conducted in the absence of any commercial or financial relationships that could be construed as a potential conflict of interest.
